# Abrasive Wear Properties of Wear-Resistant Coating on Bucket Teeth Assessed Using a Dry Sand Rubber Wheel Tester

**DOI:** 10.3390/ma17071495

**Published:** 2024-03-26

**Authors:** Zhongxin Wang, Long Sun, Dong Wang, Bo Song, Chang Liu, Zhenning Su, Chaobin Ma, Xiaoyong Ren

**Affiliations:** 1 School of Mining, Liaoning Technical University, Fuxin 123000, China; zhongxindeyouxiang@163.com (Z.W.); lntu_wd@163.com (D.W.); 2 Digital and Intelligent Industry Center, China Coal Technology Engineering Group (CCTEG), Shenyang Engineering Company, Shenyang 110013, China; songbo1984923@163.com (B.S.); iamdamei.111@163.com (C.L.); tianwangszn@gmail.com (Z.S.); 3 School of Mechanical and Electrical Engineering, China University of Mining and Technology-Beijing, Beijing 100083, China; chaobinma00@163.com

**Keywords:** abrasive wear, bucket tooth, laser cladding, hard-facing, wear mechanism

## Abstract

Ni60-WC coatings with different WC contents on the bucket tooth substrates were pre- pared using laser cladding technology. Their abrasive wear properties were assessed using the dry sand rubber wheel test system. The substrate and the hard-facing layer were tested for comparison. The results showed that the hardness of the Ni60-WC coatings increased with the increase in WC content. The wear resistance of the bucket tooth substrate was greatly improved by hard-facing and laser cladding Ni60-WC coatings. The wear rate of the hard-facing layer was reduced to 1/6 of that of the tooth substrate. The wear rate of the laser cladding coatings with 20–40 wt.% WC was similar to that of the hard-facing layer. It is worth mentioning that the wear rate of the coatings with 60–80 wt.% WC was only 1/4 of that of the hard-facing layer. Micro-cutting with surface plastic deformation was the main wear mechanism of the substrate to form narrow and deep furrows. The wear mechanism of the hard-facing layer was mainly plastic deformation with a wide groove, and the surface cracks promoted the removal of the material. The removal of the binder phase caused by micro-cutting was the main wear mechanism of the laser cladding Ni60-WC coatings. However, the hard phase of WC hinders micro-cutting and plastic deformation, which improves the wear resistance of the coating.

## 1. Introduction

Bucket teeth, which are typically positioned at the forefront of an excavator, are highly susceptible to significant and intricate wear because of the direct contact with ores, gravel, and other materials [1,2,3,4]. Their wear failure will reduce productivity and increase equipment downtime, resulting in considerable economic costs [3]. The usual wear failure forms of bucket teeth include impact, abrasion, chemical action, and fretting [1]. The most common type of wear in bucket teeth is considered to be abrasive wear, which occupies a significant portion of overall wear. Many researchers have focused on the abrasive wear behavior of bucket teeth and how to improve abrasive wear resistance over the years [5,6,7].

Several laboratory tribo-apparatuses have been designed, such as dry sand rubber wheel test (DSRWT) [8], wet sand rubber wheel test (WSRWT) [9], and sand steel wheel test (SSWT) [10], to evaluate the abrasive wear resistance of the materials, which are used in the mining, oil sand, and agricultural machinery industries [11,12,13,14,15,16,17]. These apparatuses can measure three-body wear under different service conditions. The sample is pressed against the rim of a rotating rubber or steel wheel. The abrasive sand enters the gap between the wheel and the sample, producing wear on the sample under an applied normal load at a certain sliding speed. The abrasion resistance of a material is evaluated by measuring its volume loss after the test. The DSRWT is one of the most effective testing methods for the bucket tooth materials according to their usual service condition [18].

The most common mean is to prepare a protective coating on the surface to improve the wear resistance of the bucket tooth materials [19]. Hardfacing is a common and economical way to prepare a wear-resistant coating. The service life and efficiency of wearing metal parts are improved by forming a protective coating of the proper metallurgy during hardfacing [20,21,22]. Laser cladding technology is a recently developed surface coating method for improving the wear resistance of alloys [23,24,25,26,27]. The laser beam melts the powder material on the surface to form a fully dense, metallurgically bonded coating [28]. The wear-resistant coating prepared using laser cladding technology is expected to further improve the wear resistance of bucket teeth [27]. Ni-based alloys have been widely used as blades, bearings, and valves [29]. Sahu prepared Ni_1−x_Ti_x_N films using magnetron co-sputtering, and the hardness and other friction properties of the films can be improved with the increase in TiN content [30]. A great deal of attention has been paid to the preparation of Ni60 coating using laser cladding [5,6]. However, with the improvement in the service life of bucket teeth, the wear resistance of the coating needs to be further improved. The composite coating formed by Ni60 and other materials has attracted a lot of attention [31,32].

In this work, the coatings with Ni60 as matrix containing different contents of WC on the bucket tooth material were prepared using laser cladding technology. The wear characteristics and wear resistance of the coating were assessed using the dry sand rubber wheel test system according to the ASTM G65 standard. As a comparison of wear resistance, the same tests were performed on the substrate and hard-facing coating of the bucket teeth. The wear rate and wear mechanism of the specimen were studied, which can then be used to predict the service life of the bucket teeth. The selection of wear-resistant material should consider the operational environment and specific types of abrasive material [33]. Considering the working environment of the bucket teeth, quartz sand was chose as the abrasive.

## 2. Materials and Methods

### 2.1. Test System and Procedures

**Figure 1:**
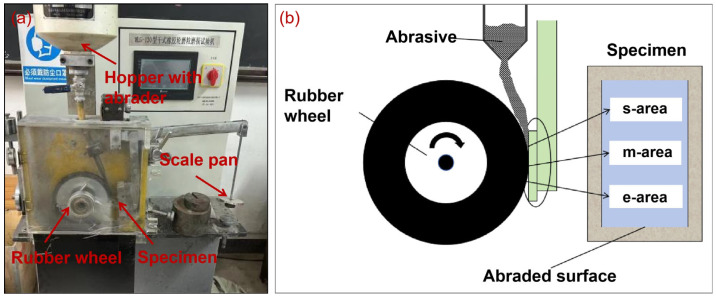
The real image of the MLG-130A dry sand rubber wheel test system (**a**) and the schematic presentation of the test method (**b**).

**Figure 2:**
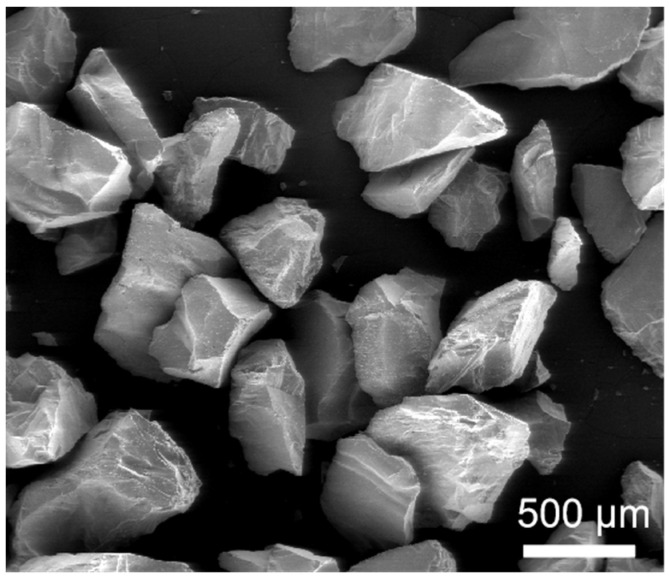
Typical SEM image of the quartz sand used in this work.

The experimental tests were performed on a dry sand rubber wheel test system (MLG 130A, Zhangjiakou, China), according to a modified ASTM G65 standard [8]. Figure 1 shows the real image of the MLG-130A dry sand rubber wheel test system and the schematic presentation of the test method. The abraded surface of the samples was divided into starting area (s-area), middle area (m-area), and ending area (e-area), according to the order of contact with the sand particles, to facilitate the wear mechanism. The rubber wheel used in this work has a shore hardness of 60 degrees and a diameter of 229 mm. The abrasives used in this work was coarse quartz (SiO_2_, white) sand. Figure 2 shows the typical SEM image of the angular quartz sand. It can be seen that the quartz is coarsely angular, with an average size of about 500 μm. The spike parameter quadratic (SPQ) value of the quartz sand is about 0.561, and the hardness of the abrasive is about 1100 HV.

During the testing, the sample is pressed against the rubber wheel under a weight-load. The quartz sand enters the gap between the sample and the rubber wheel. The sample is pre-ground for 100 revolutions under a load of 20 N to reduce the differential due to the roughness of the machined surface. After the pre-grinding, the sample is cleaned in absolute ethyl alcohol and weighed for the basic mass. Then, the sample is tested under a load of 80 N at a rotation speed of 120 rpm. The sample is weighed after 1000 revolutions of grinding to calculate the wear loss.

### 2.2. Materials

A rectangular block (57 × 25.5 × 4 mm^3^) cut from the bucket teeth by wire-electrode cutting was used as the substrate. The oxide layer on the surface of the substrate was removed by the grinder before laser cladding. Then, ultrasonic cleaning was carried out to ensure that the surface of the substrate was clean. Commercially Ni60 and WC powders with a average size of 50 μm were used as the laser cladding powder. Before laser cladding, the powder was milled and mixed using a planetary ball mill. Then, the powder was dried using a blast drying box to ensure the dryness and fluidity of the powder. The laser cladding materials were the combination of a Ni60 alloy powder with a particle size distribution of about 50–150 μm and a WC powder with a size of 45–106 μm. The chemical composition of the Ni60 alloy powder was 0.6–1.0 wt.% C, 14–17 wt.% Cr, 2.5–4.5 wt.% B, 3–4.5 wt.% Si, and <15 wt.% Fe; Ni constituted the balance. The laser cladding processing was carried out using MobiMRO-12F-600 Laser cladding equipment (Huirui, Tianjin, China). Before the cladding process, the substrate needs to be preheated at 180–200 ℃ for one hour. Laser cladding was performed using a laser power of 1300 W, a scanning speed of 8 mm/s, a powder feeding rate of 10 g/min, a spot diameter of 2.2 mm, and a overlapping rate of 40%.

**Figure 3:**
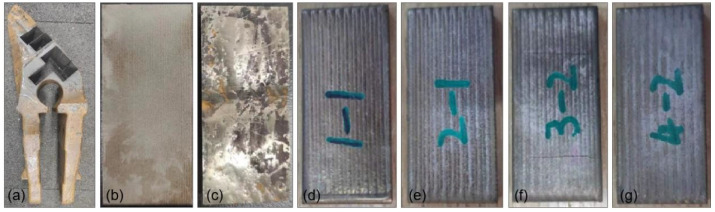
Typical macroscopic images of the specimens: (**a**) bucket tooth after wire-electrode cutting, (**b**) substrate, (**c**) hardfacing specimen, and (**d**–**g**) laser cladding specimens.

**Figure 4:**
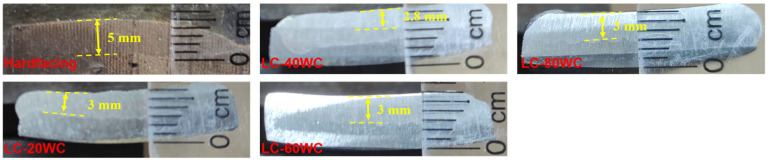
The cross-section images of the hardfacing sample and laser cladding samples with different contents of WC addition.

Figure 3a shows the real image of the bucket tooth after wire-electrode cutting. The macroscopic morphology of the substrate and hardfacing samples obtained by cutting is shown in Figure 3b,c, and the corresponding chemical compositions are given in Table 1. The wear-resistant coating was prepared on the substrate surface using laser cladding technology. The designed compositions of the coating are shown in Table 1, and the macroscopic morphology of the sample after cladding is shown in Figure 3d,g. Figure 4 shows the cross-section images of the hardfacing sample and laser cladding samples with different contents of WC addition. The thickness of the hardfacing layer was about 5 mm, and the thickness of the laser cladding coatings was about 2.8~3 mm.

**Figure 5:**
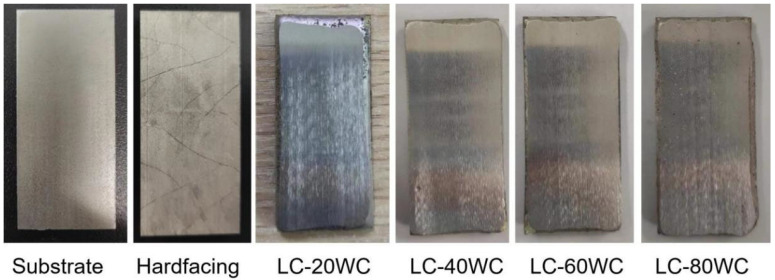
Typical macroscopic images of the specimens before dry sand rubber wheel tests.

**Figure 6:**
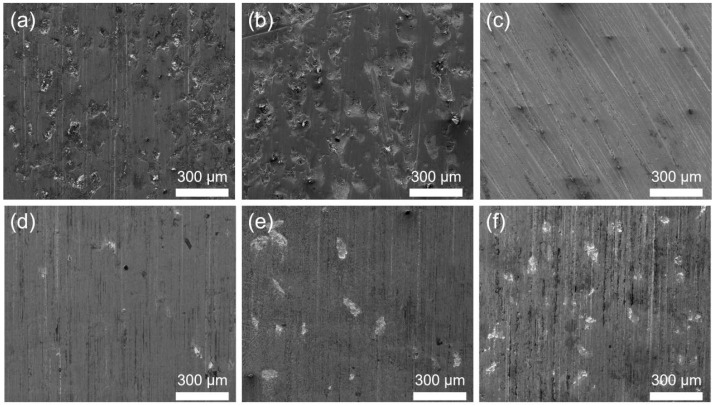
Typical SEM images of the surface of the specimens before dry sand rubber wheel tests: (**a**) substrate, (**b**) hardfacing, (**c**) LC-20WC, (**d**) LC-40WC, (**e**) LC-60WC, and (**f**) LC-80WC.

**Figure 7:**
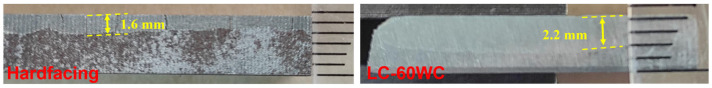
The cross-section images of the hardfacing and laser cladding samples after grinding and polishing.

All samples were smoothed using a grinder equipped with a diamond wheel before the experiment to avoid the experimental error caused by the uneven surface of the samples. The macroscopic images and SEM images of the smoothed samples are shown in Figure 5 and Figure 6. It can be seen that there are many cracks on the surface of the hardfacing sample, but no cracks appeared on the laser cladding coating surface. Figure 7 shows the cross-section images of the hardfacing and laser cladding samples after grinding and polishing. There are a few cracks in the hardfacing layer, and most cracks go deep into the substrate. The laser cladding coating is relatively dense, and no obvious cracks are observed in the cross-section image. Both the hardfacing layer and laser cladding coating are closely bonded to the substrate.

### 2.3. Characterization

The wear volume loss of the sample after the test was calculated by measuring the mass loss using an analytical balance (FA2204E, Hengping, Shanghai, China) with an accuracy of 0.001 g. The wear rate of the material in this work was calculated by calculating the wear volume loss per unit wear distance. The hardness of the samples was measured using a Vickers hardness tester (TuKon2500B, Wilson, Chicago, IL, USA) with a loading of 1 kgf for 15 s. The measurements of the mass and hardness in this work were tested five times. The average value was taken as the corresponding mass and hardness values. The phase composition of the coatings was analyzed using X-ray diffraction (XRD, D8-Advance, Bruker, Saarbrucken, Germany) with Cu Kα radiation (λ = 1.54178 Å) through a continuous scanning mode at a speed of 5 °/min. The microstructures of the samples were observed using an optical microscope (OM, VHX-5000, Keyence, Osaka, Japan) and a scanning electron microscope (SEM, Quanta 200 FEG, FEI, Hillsboro, OR, USA). The elemental composition was measured using an EDX spectrometer mounted on the SEM. The wear surfaces on each sample were analyzed using a 3D white-light interferometer (Nexview, Zygo Lamda, Middlefield, CT, USA).

## 3. Results and Discussion

### 3.1. Microstructure, Phase Composition, and Microhardness

**Figure 8:**
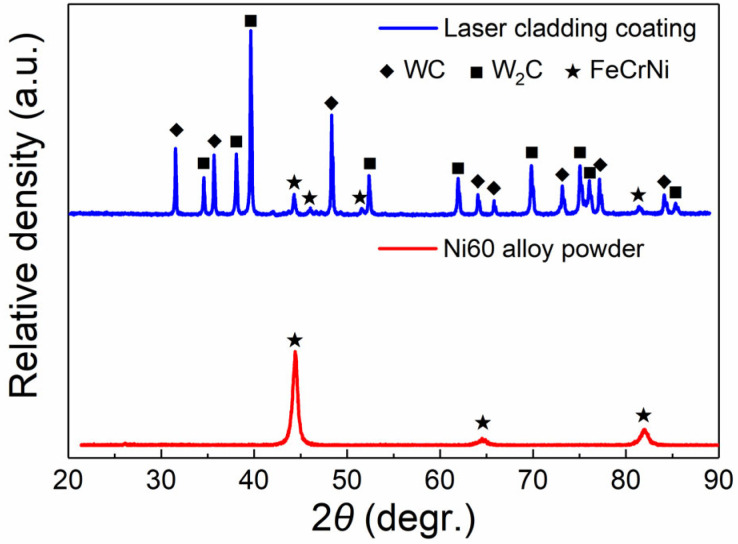
XRD patterns of the Ni60 alloy powder and LC-80WC laser cladding coating.

Figure 8 shows the phase evaluation of the coating after laser cladding. It can be seen that the phase of the Ni60 alloy powder is mainly the peak of NiCrFe (PDF card: 35-1375). For the laser cladding coating with 80 wt.% WC, peaks of WC (PDF card: 65-8828) and W_2_C (PDF card: 65-3896) are also detected besides the NiCrFe phase. According to the SEM images on the surface and the XRD analysis, it can be inferred that most of the WC is retained, while some WC is decarbonized in the laser cladding process, forming W_2_C.

**Figure 9:**
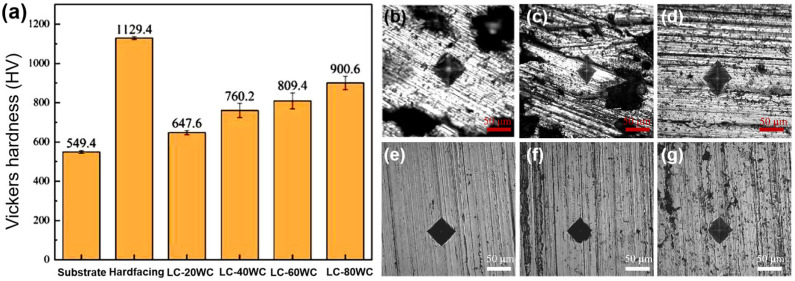
Vickers hardness of the samples (**a**) and the corresponding indentation images: (**b**) substrate, (**c**) hardfacing, (**d**) LC-20WC, (**e**) LC-40WC, (**f**) LC-60WC, and (**g**) LC-80WC.

Figure 9 illustrates the Vickers hardness values and the corresponding indentations measured from the coating surface of the samples. Each sample was tested five times to get an average value. The average hardness values of the substrate and hardfacing layer are 549 and 1129 HV_1.0_, respectively. The hardness of the hardfacing layer is twice higher than that of the substrate, which is beneficial to the improvement of the wear resistance of the sample. The increase in hardness has been attributed to the surface alloying of chromium. The hardness of the laser cladding layers gradually increased from 647 HV_1.0_ to 900 HV_1.0_ with the contents of WC increasing from 20 wt.% to 80 wt.%. As a hard reinforcing phase, the increase of WC is conducive to the increase of the coating hardness. The coating is a composite of Ni60 and WC, which is similar to cemented carbide. The hardness of the laser cladding coating in this work is lower than that of the hardfacing layer, but its wear resistance is expected to be effectively improved because of the existence of WC.

### 3.2. Dry Sand Rubber Wheel Test

**Figure 10:**
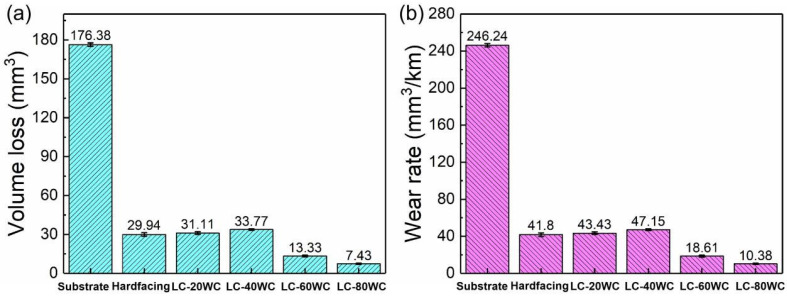
Wear volume (**a**) and wear rate (**b**) of the samples.

Figure 10 shows the dry sand rubber wheel test results in the form of wear volume loss and wear rate. As indicated by the results, the substrate presented the highest wear volume and wear rate of 176.38 mm^3^ and 246.25 mm^3^/km, respectively. The wear rate of the hardfacing layers is much lower than that of the substrate, only 1/6 of that of the substrate, which is mainly attributed to the increase in the hardness of the hardfacing layer. The results also prove that hardfacing is an effective way to improve the wear resistance of bucket teeth. The wear volumes and rates of the laser cladding coatings with the addition of 20 wt.% and 40 wt.% WC were close to those of the hardfacing layer, although the hardness of both laser cladding samples was much lower than that of the hardfacing sample. The results indicated that the wear forms of the Ni60-WC composite coating prepared using laser cladding are different from that of the hardfacing layer. Also, they prove that the hard phase played a certain role in the improvement in wear resistance. With the increase in WC content, the wear volume and wear rate of the laser cladding coating gradually decreased, reaching the minimum values of 7.43 mm^3^ and 10.38 mm^3^/km with 80 wt.% WC addition. The wear rate of the coating with 80 wt.% WC addition is about 24 times lower than that of the substrate and also about 4 times lower than that of the hardfacing samples. The results mean that the Ni60-WC composite coating prepared using laser cladding showed excellent wear resistance.

### 3.3. Surface Morphology after Wear Process

**Figure 11:**
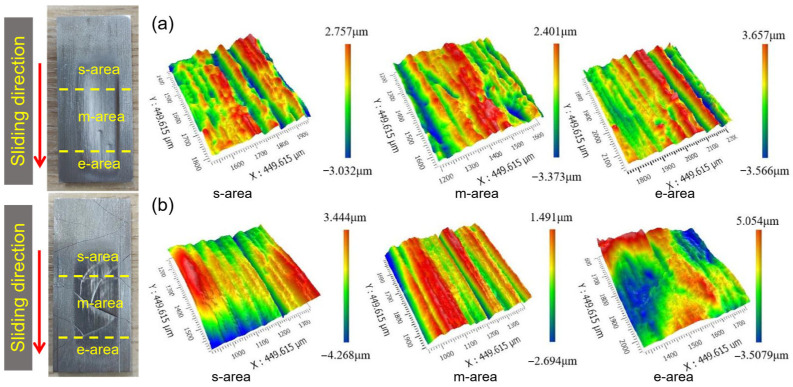
Typical photos and 3D white-light interferograms of the abraded surface of the substrate (**a**) and hardfacing layer (**b**) after the dry sand rubber wheel tests.

Figure 11 shows the typical photos and 3D white-light interferograms of the abraded surface of the substrate and hardfacing layer after the dry sand rubber wheel tests. The abraded surface was divided into starting area (s-area), middle area (m-area), and ending area (e-area) according to the order of contact with the sand particles. During the wear process, the sand particle entered the contact area between the rubber wheel and the sample from the s-area, forming three-body wear. Then, the sand particle was driven into the m-area under the rotation of the rubber wheel. In this process, the contact load between the sand particle and the sample gradually increased, reaching the maximum in the middle part. Therefore, the m-area displayed the most severe wear and the deepest wear marks. Finally, the sand slipped to the e-area and then separated from the sample. In this process, the contact load between the sand particle and the sample gradually decreased and some of the sand particles were broken.

Compared with the wear surface morphology of each sample, the wear marks of the substrate were the deepest, corresponding with the largest wear volume. There are many cracks on the surface of the hardfacing layer and the cracks became wider after the test. Furrows could be observed on the wear surface of the laser cladding coating and there were many pits in the area of abrasion marks.

**Figure 12:**
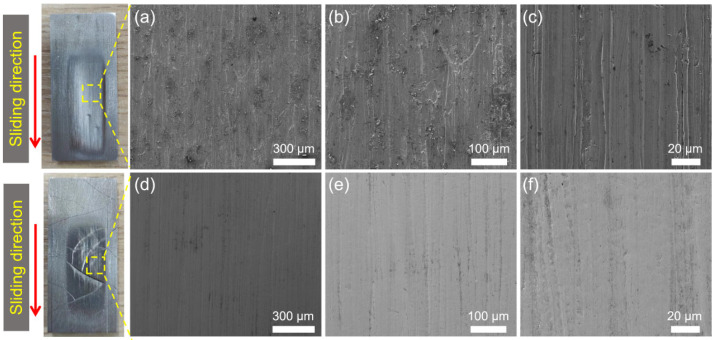
Typical photos and SEM images of the abraded areas of the substrate (**a**–**c**) and hard-facing layer (**d**–**f**) after the dry sand rubber wheel tests. The red arrow represents the sliding direction during the wear test.

Figure 12 gives the typical SEM images of the abraded surface morphologies of the substrate and the hardfacing layer. A variety of typical abrasive wear mechanisms, such as plastic deformation, micro-cutting, and grain fracture, can be seen on the wear surface of the samples. However, the main wear mechanism of the samples is different. Because the hardness of the substrate is much lower than that of the quartz sand, the sharp quartz particle can easily be inserted into the substrate surface. Therefore, micro-cutting is the main wear mechanism for the substrate. The furrows and scratches caused by micro-cutting can be seen in Figure 12a–c. This is also the main reason why the substrate samples show the highest wear rate.

**Figure 13:**
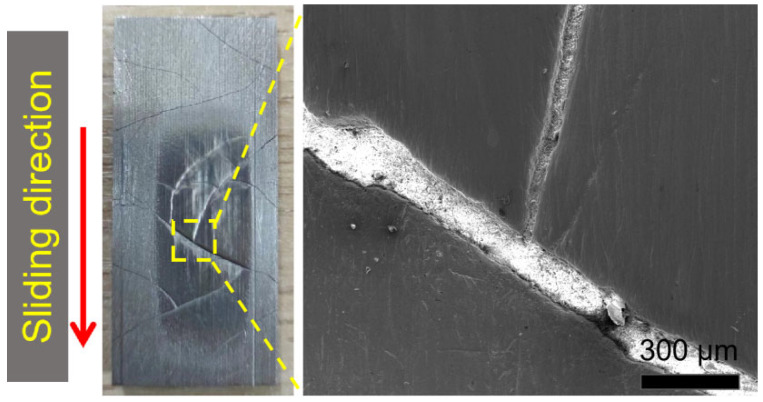
Typical photo and SEM image of the crack wear area of the hard-facing layer. The red arrow represents the sliding direction during the wear test.

The wear morphology of the hard-facing layer is a relatively wide and smooth groove formed by plastic deformation. That means that plastic deformation play the main role in the wear mechanism of the hard-facing layer sample. This is mainly because the hardness of the hard-facing layer is similar to that of the quartz sand. It is not easy for the abrasive particles to be directly inserted into the sample surface to form micro-cutting, but they are more inclined to cause plastic deformation under the action of the load. After the repeated extrusion and plastic deformation of the samples, a wide and smooth groove is formed, which also caused the fatigue removal of the sample during this process. In abrasive wear, the material removal caused by plastic deformation is much lower than that of micro-cutting [11,33], so the wear rate of the hard-facing layer is much lower than that of the substrate, only one-sixth of it. However, due to the existence of a large number of cracks in the hard-facing layer (Figure 13), the wear chips would continuously invade the cracks during the wear process, which would aggravate the wear of the hard-facing layer.

**Figure 14:**
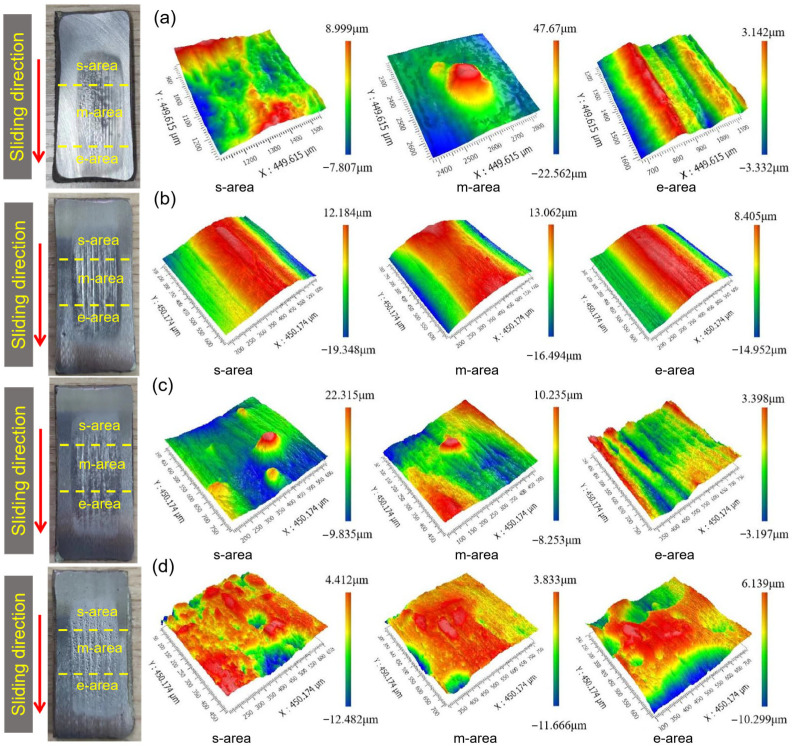
Typical photos and 3D white-light interferograms of the abraded surface of the laser cladding coating with different WC addition: (**a**) 20 wt.%, (**b**) 40 wt.%, (**c**) 60 wt.%, and (**d**) 80 wt.%. The red arrow represents the sliding direction during the wear test.

**Figure 15:**
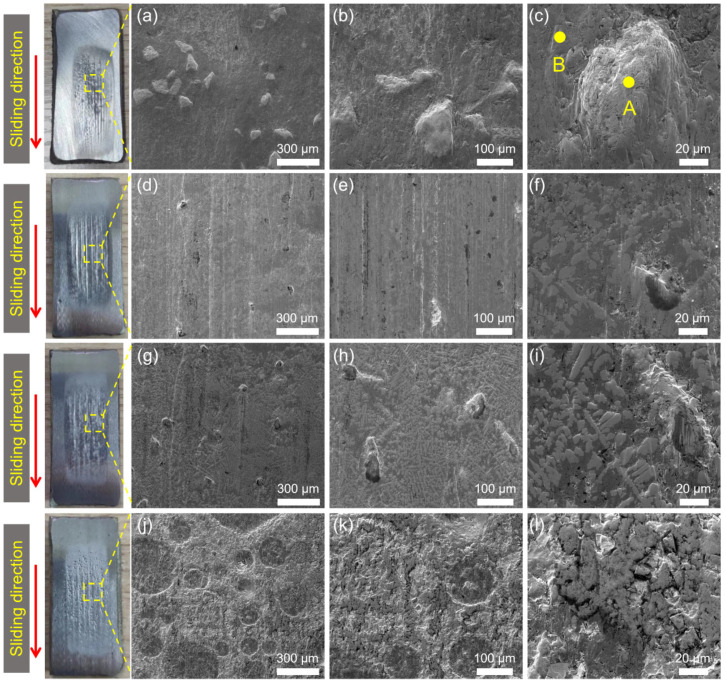
Typical photos and SEM images of the abraded areas of t of the laser cladding coating with different WC addition: (**a**–**c**) 20 wt.%, (**d**–**f**) 40 wt.%, (**g**–**i**) 60 wt.%, and (**j**–**l**) 80 wt.%. The red arrow represents the sliding direction during the wear test. The points A and B in subgraph (**c**) refer to the positions where the EDS is tested in Table 2.

Figure 14 and Figure 15 show the typical photos, 3D white-light interferograms, and SEM images of the abraded surface of the laser cladding coating with different WC addition. Several furrows caused by the micro-cutting can be observed on the laser cladding coatings with 20 wt.% WC addition. There are some protrusions in the furrow, which block the micro-cutting. The hardness of the coating with 20 wt.% WC is similar to that of the substrate. Therefore, during the wear process, sharp sand particles can be easily inserted into the surface, resulting in micro-cutting. However, the wear rate of the laser cladding coating with even 20 wt.% WC is much lower than that of the substrate. The improvement in the wear resistance of the laser cladding coating is mainly attributed to the hindering effect of the protrusions in the furrow on micro-cutting. Energy-dispersive spectrum analysis was carried out to confirm the composition of the protrusions. The analysis result is shown in Table 2, and the corresponding detected area is shown in Figure 15c. The main elements of the protrusions are C, W, and O. The atomic percentage of C and W is close to 1:1, indicating that the protrusion is mainly WC hard phase. This proves that the wear resistance of the laser cladding coating can be further improved by adding WC hard phase.

With the increase in the content of the WC addition, the hardness of the laser cladding coatings gradually increased, as shown in Figure 9. It can be seen that with the increase in WC content, the furrows in the abrasion marks were no longer continuous and were blocked by the increasing WC protrusions. Even when the WC content reaches 80%, the hard WC protrusions occupied most of the proportion of the abrasion surface. At this time, the wear morphology was mainly observed in the removal of adhesive phase to form the pits.

The hardness of all laser cladding coating samples is lower than that of the hardfacing layer in this work, even if the coating had an 80 wt.% WC addition. However, the wear rate of the laser cladding coating is much lower than that of the hard-facing layer, reducing to 1/2 and 1/4 with 60 wt.% and 80 wt.% WC addition, respectively. This is mainly due to the increase in WC, which makes it difficult for the sharp quartz sand to form serious micro-cutting and plastic deformation. The WC grains breaking and pulling out gradually became the main wear mechanism. The hardness and strength of WC are much higher than that of the quartz sand, so it is difficult to break WC grains during the abrasive wear process. At the same time, The WC particles and Ni60 matrix bonded together tightly by using laser cladding technology. It is difficult to pull out WC grains during wear, resulting in the great improvement of the laser cladding coating.

### 3.4. Wear Mechanism

**Figure 16:**
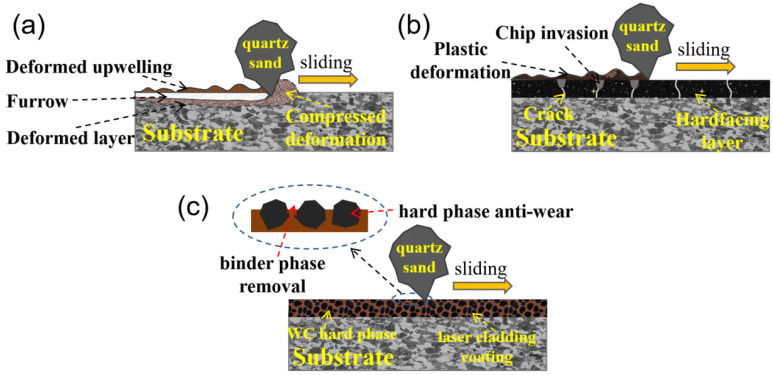
Schematic description of the different abrasive wear mechanisms of the substrate (**a**), hardfacing layer (**b**), and laser cladding coating (**c**).

The different abrasive wear mechanisms of the substrate, hardfacing layer, and laser cladding coating in this work are summarized in Figure 16. Due to the relative low hardness of the substrate, the sharp quartz sand could easily be inserted into the surface. The main wear mechanism was micro-cutting to form furrows, accompanied by plastic deformation, as shown in Figure 16a. The quartz sands were driven into the gaps between the rubber wheel and the samples in the s-area, and the furrows gradually formed in the m-area. At last, the sand was driven out from the e-area and separated from the sample. The m-area displayed the deepest furrow and the most serious wear, as shown in Figure 11a.

Because the hardness of the hardfacing layer was close to that of the quartz sand, it was difficult for quartz sand to penetrate the surface of the hardfacing layer. At that time, the main wear mechanism was surface plastic deformation, as shown in Figure 12b. At the same time, because there were a large number of cracks on the surface of the hardfacing layer, broken quartz sand and abrasive chips gradually invaded the cracks, expanding the cracks and causing the wear of the surface.

For laser cladding Ni60-WC composite coating, the main wear mechanism was binder phase removal caused by micro-cutting and plastic deformation. The WC bonded in the coating was like a hard obstacle, hindering micro-cutting and plastic deformation, and improving the wear resistance of the samples. With the increase in WC content in the coating, the obstruction wear effect of WC barriers was more obvious. Therefore, within the scope of WC addition in this work, the more WC is added, the better the wear resistance of the laser cladding coating.

## 4. Conclusions

In this work, the abrasive wear properties of laser cladding coating with Ni60-WC were assessed using the dry sand rubber wheel test system. Accordingly, the substrate and the hardfacing layer were tested for wear resistance comparison. The main conclusions are drawn as follows.

1. The Ni60-WC composite coatings were successfully prepared using laser cladding technology, and the coating’s hardness increased with the increase in WC content, reaching the maximum value of 900 HV with an 80 wt.%WC addition.
2. Using hardfacing, the wear resistance of bucket teeth can be greatly improved, and the wear rate can be reduced to 1/6 of the tooth substrate. The wear resistance of the bucket teeth can also be greatly improved using laser cladding Ni60-WC coatings. The wear rate of the coatings with 20–40 wt.% WC was similar to that of hardfacing layer. The wear resistance of the coatings with 60–80 wt.%WC was 4 times higher than that of the hardfacing layer and 24 times higher than that of the bucket tooth substrate.
3. The abrasive wear mechanism of the bucket tooth substrate was mainly micro-cutting with surface plastic deformation, forming narrow and deep furrows. The wear mechanism of hardfacing layer was mainly plastic deformation, forming a wide groove. At the same time, the cracks on the hardfacing layer promoted the removal of the material. The main wear mechanism of the laser cladding coating was the removal binder phase caused by micro-cutting. However, the hard phase of WC hindered micro-cutting and plastic deformation, which improved the wear resistance of the coating.

## Figures and Tables

**Table 1:** Chemical and designed compositions of the samples in this work.

Samples No.	Compositions (wt.%)
Substrate	C: 0.26; Mn: 0.93; Si: 1.39; Mo: 0.28; Ni: 0.36; Cr: 1.68; Fe: Bal.
Hardfacing	C: 0.13; Mn: 1.11; Si: 0.76; Ti: 0.1; Ni: 0.092; Cr: 10.52; Fe: Bal.
LC-20WC	80 wt.% Ni60 + 20 wt.% WC
LC-40WC	60 wt.% Ni60 + 40 wt.% WC
LC-60WC	40 wt.% Ni60 + 60 wt.% WC
LC-80WC	20 wt.% Ni60 + 80 wt.% WC

**Table 2:** Main element content of different positions on the abraded surface of the laser cladding coating, as shown in the SEM image of Figure 15c.

Elements	C	O	W	Si	Cr	Fe	Ni	Total
Point A (at.%)	45.6	10.7	43.7	-	-	-	-	100
Point B (at.%)	18.7	3.2	2.0	3.9	33.2	12.2	26.8	100

## Data Availability

All data needed to evaluate the conclusions in the paper are present in the paper. Additional data related to this paper may be requested from the authors.

## References

[1] Dong Z., Jiang F., Tan Y., Wang F., Ma R., Liu J. (2023). Review of the Modeling Methods of Bucket Tooth Wear for Construction Machinery. Lubricants.

[2] Singla S., Kang A.S., Grewal J.S., Cheema G.S. (2014). Wear Behavior of Weld Overlays on Excavator Bucket Teeth. Procedia Mater. Sci..

[3] Filip M., Predrag J., Djenadića S. (2018). Behavior Determining of Bucket Wheel Drive Depending on the Wear Impact of the Cutting Elements. Procedia Struct. Integr..

[4] Rusiński E., Cegiel L., Michalczyk A., Moczko P., Olejarz J., Pietrusiak D. (2015). Investigation and modernization of buckets of surface mining machines. Eng. Struct..

[5] Lu P., Jia L., Zhang C., Heng X., Xi X., Duan M., Lu Z., Zhou Y. (2023). Optimization on laser cladding parameters for preparing Ni60 coating along with its friction and wear properties. Mater. Today Commun..

[6] Wang C., Gao Y., Wang R., Wei D., Cai M., Fu Y. (2018). Microstructure of laser-clad Ni60 cladding layers added with different amounts of rare-earth oxides on 6063 Al alloys. J. Alloys Compd..

[7] Keleş A., Yildirim M. (2020). Improvement of mechanical properties by means of titanium alloying to steel teeth used in the excavator. Eng. Sci. Technol. Int. J..

[8] (2010). Standard Test Method for Measuring Abrasion Using the Dry Sand/Rubber Wheel Apparatus.

[9] (2007). Standard Test Method for Conducting Wet Sand/Rubber Wheel Abrasion Tests.

[10] (2005). Standard Test Method for Abrasive Wear Resistance of Cemented Carbides.

[11] Ren X., Peng Z., Hu Y., Rong H., Wang C., Fu Z., Qi L., Miao H. (2014). Three-body abrasion behavior of ultrafine WC-Co hardmetal RX8UF with carborundum, corundum and silica sands in water-based slurries. Tribol. Int..

[12] Yue C., Cai H., Kong L., Liang C., Peng Z., Wang Y. (2022). Wear Behaviors of AISI 4145H Drilling Tool Steel under Drilling Fluid Environment Conditions. Materials.

[13] Sanna A.K., Päivi K.R., Jari L., Jussi H., Simo-Pekka H. (2007). Abrasive wear properties of tool steel matrix composites in rubber wheel abrasion test and laboratory cone crusher experiments. Wear.

[14] Bialobrzeska B., Jasinski R. (2023). Resistance to Abrasive Wear with Regards to Mechanical Properties Using Low-Alloy Cast Steels Examined with the Use of a Dry Sand/Rubber Wheel Tester. Materials.

[15] Yousif B.F. (2013). Design of newly fabricated tribological machine for wear and frictional experiments under dry/wet condition. Mater. Des..

[16] Elalem K., Li D.Y. (2001). Variations in wear loss with respect to load and sliding speed under dry sand/rubber-wheel abrasion condition: A modeling study. Wear.

[17] Rajendhran N., Pondicherry K., Huang S., Vleugels J., Sukumaran J., De Baets P. (2024). Influence of grit particles characteristics on the abrasive wear micro-mechanisms of NbC-Ni and WC-Co hard materials. Int. J. Refract. Met. Hard Mater..

[18] Katinas E., Chotěborský R., Linda M., Kuře J. (2021). Sensitivity analysis of the influence of particle dynamic friction, rolling resistance and volume/shear work ratio on wear loss and friction force using DEM model of dry sand rubber wheel test. Tribol. Int..

[19] Yakovlev A., Bertrand P., Smurov I. (2004). Wear-resistant coatings with engineered structure by laser cladding. Tribol. Lett..

[20] Liu W., Gao D. (2021). Microstructure and wear of Ni-WC hardfacing used for steel-body PDC bits. Int. J. Refract. Met. Hard Mater..

[21] Kumar S., Mondal D.P., Jha A.K. (2000). Effect of Microstructure and Chemical Composition of Hardfacing Alloy on Abrasive Wear Behavior. J. Mater. Eng. Perform..

[22] Brezinová J., Draganovská D., Guzanová A., Balog P., Viňáš J. (2016). Influence of the Hardfacing Welds Structure on Their Wear Resistance. Metals.

[23] Tuominen J., Kiviö J., Balusson C., Raami L., Vihinen J., Peura P. (2023). High-speed laser cladding of chromium carbide reinforced Ni-based coatings. Weld. World.

[24] Wang S., Chen Y., Gu C., Sai Q., Lei T., Williams J. (2023). Antifouling Coatings Fabricated by Laser Cladding. Coatings.

[25] Wang C., Gao Y., Zeng Z., Fu Y. (2017). Effect of rare-earth on friction and wear properties of laser cladding Ni-based coatings on 6063Al. J. Alloys Compd..

[26] Nurminen J., Näkki J., Vuoristo P. (2009). Microstructure and properties of hard and wear resistant MMC coatings deposited by laser cladding. Int. J. Refract. Met. Hard Mater..

[27] Gu Z., Xi S., Sun C. (2020). Microstructure and properties of laser cladding and CoCr2.5FeNi2Tix high-entropy alloy composite coatings. J. Alloys Compd..

[28] Li J., Ju J., Chang W., Yang C., Wang J. (2020). Investigation on the Microstructure and Wear Behavior of Laser-Cladded High Aluminum and Chromium Fe-B-C Coating. Materials.

[29] Yang X., Wang X., Zhou J., Wei H., Zeng R., Li W. (2023). Effect of Cu addition on the microstructure and tribological performance of Ni60 directional structure coating. Int. J. Miner. Metall. Mater..

[30] Sahu M.M.R. (2020). Structure and properties of Ni_1–X_Ti_X_N thin films processed by reactive magnetron co-sputtering. Mater. Charact..

[31] Yang X., Li X., Yang Q., Wei H., Fu X., Li W. (2020). Effects of WC on microstructure and corrosion resistance of directional structure Ni60 coatings. Surf. Coat. Technol..

[32] Liao H., Normand B., Coddet C. (2000). Influence of coating microstructure on the abrasive wear resistance of WC/Co cermet coatings. Surf. Coat. Technol..

[33] Szala M., Szafran M., Matijošius J., Drozd K. (2023). Abrasive Wear Mechanisms of S235JR, S355J2, C45, AISI 304, and Hardox 500 Steels Tested Using Garnet, Corundum and Carborundum Abrasives. Adv. Sci. Technol. Res. J..

